# Bioactive Constituents of *Lamium album* L. as Inhibitors of Cytokine Secretion in Human Neutrophils

**DOI:** 10.3390/molecules23112770

**Published:** 2018-10-25

**Authors:** Monika E. Czerwińska, Anita Świerczewska, Sebastian Granica

**Affiliations:** Department of Pharmacognosy and Molecular Basis of Phytotherapy, Medical University of Warsaw, 1 Banacha street, 02-097 Warsaw, Poland; anita_sw@wp.pl (A.S.); sgranica@wum.edu.pl (S.G.)

**Keywords:** phenylpropanoids, iridoids, flavonoids, Lamiaceae, cytokines, inflammation

## Abstract

The traditional role of *Lamium album* L. (white dead nettle, Lamiaceae) in providing relief from pain in rheumatism as well as vaginal and cervical inflammation was described. The aim of the study was to screen for the anti-inflammatory bioactivity of compounds isolated from aqueous-methanolic extract of *Lamium album* herb in human neutrophils (PMNs). The effect of the compounds on the inhibition of selected inflammatory markers released by neutrophils, such as cytokines (IL-8, TNF-α), was studied. The molecular masses and the purity of compounds were determined using high-performance liquid chromatography coupled with diode array detection and mass spectrometry (HPLC-DAD-MS^n^). The level of cytokines production after incubation with the compounds (1–25 µM) was measured by ELISA. Two derivatives of quercetin, not previously described, were isolated in this study. Phenylpropanoids (verbascoside and phlinoside D), as well as iridoids (lamalbid, and shanzhiside methyl ester), and flavonoids revealed to be more significant inhibitors of IL-8 secretion than TNF-α. The compounds at a concentration of 25 µM, except for shanzhiside methyl ester (**6**), inhibited secretion of IL-8 in the range from 29.1 to 50.0%. In conclusion, *L. album* might be a valuable source of bioactive compounds and may provide constituents to limit noninfectious inflammation associated with the aforementioned diseases.

## 1. Introduction

The traditional role of *Lamium album* L. (white dead nettle, Lamiaceae) in providing pain relief from rheumatism as well as vaginal and cervical inflammations was described [1]. However, the aerial parts of white dead nettle have also been used as alternative nourishment during the starvation seasons in different countries of Asia and Europe, in particular, in Turkey, China, and Japan [2,3]. The leaves and flowers are edible and can be consumed raw or cooked, and they are a spice and/or specific ingredient of some Mediterranean dishes [4]. Some examples of dishes: “White Dead Nettle Frittata”, “White Dead Nettle Feta and Watermelon Salad” and “Dead nettle soup” [5]. Nowadays, the infusions and food supplements enriched with *L. album* are believed to detoxify the organism, to prevent menstrual disorders and abdominal inflammation, as well as improve lipid metabolism [6]. Despite folk reports, the role of *L. album* in nutrition and traditional medicine seems to be neglected in many regions where it is widespread.

Compounds derived from phenylalanine and/or tyrosine, including phenylpropanoids, are the most common of all secondary metabolites in plants. The plant shikimate pathway is the first step of the biosynthesis of phenylpropanoids, which engages phenylalanine and tyrosine forming cinnamic acid via aromatic amino acid lyases [7,8]. Phenylpropanoids usually contain one or several C_6_–C_3_ fragments. Due to the large diversity of their structures, the classification of phenylpropanoids includes simple phenylpropanoids, such as derivatives of cinnamyl alcohols, cinnamic acids, phenylpropanes, and complex phenylpropanoids, including phenylpropanoid glycosides with the phenylethane moiety and oxidative coupling products (lignoides) [9]. The most common phenolic acids derived from cinnamic acid are *p*-coumaric, caffeic, ferulic, and sinapic acids [8,10]. In general, phenylpropanoic acids occur rarely in the free state and are very often found in esterified form, bound to carbohydrates. Thus, the group of phenylpropanoid derivatives consists particularly of phenylpropanoid esters of a glycoside comprising an oligosaccharide, such as di- or trisaccharides, and a dihydroxyphenylethanol moiety (C_6_–C_2_). These compounds can be often found in plants belonging to Lamiales, Oleales, and to a lesser extent, in the Astrales [10].

On the other hand, iridoids very often coexist with phenylpropanoids in plants. They belong to a class of monoterpenes characterized by a cyclopenta[c]pyranoid skeleton (iridane skeleton). The majority of iridoids occur as glucosides. The glycosidic linkage between the hydroxyl group of the anomeric carbon of D-glucose and the hydroxyl group in the C_1_ of the aglycone is often found. The group is considered a chemotaxonomic marker of Lamiales, Scrophulariales, and Gentianales [10].

Three phenylpropanoid glycosides, such as verbascoside, *cis*-acteoside, and lamalboside (lamiuside A) were primarily reported in *Lamium album* L. [11]. Our recent study showed the presence of others phenylpropanoids such as lamiusides B, C, and E in this species [12]. Among iridoids, lamiridoside, lamiol, caryoptoside, as well as albosides A and B were identified [13,14]. However, the bioactivity of the pure compounds of *Lamium* sp. have not been widely studied so far.

For this reason, bearing in mind that dietary supplements and nutritive constituents may affect certain human health conditions, we considered the potential role of the *L. album* herb as a source of bioactive natural products. Our aim was to screen the activity of its constituents, in particular, those belonging to the groups of phenylpropanoids, iridoids, and flavonoids, using the model of human polymorphonuclear leukocytes (PMNs, neutrophils). Leukocytes, like neutrophils, actively participate in inflammation as well as its resolution via oxidative burst and cytokine secretion. Due to the fact that overstimulation or extensive chemotaxis of leukocytes are likely to initiate and perpetuate further noninfectious inflammatory response, the study was aimed at determining the role of white dead nettle constituents in the limitation of cytokine production (IL-8, TNF-α) by PMNs.

## 2. Results

### 2.1. Identification of Compounds

**Figure 1:**
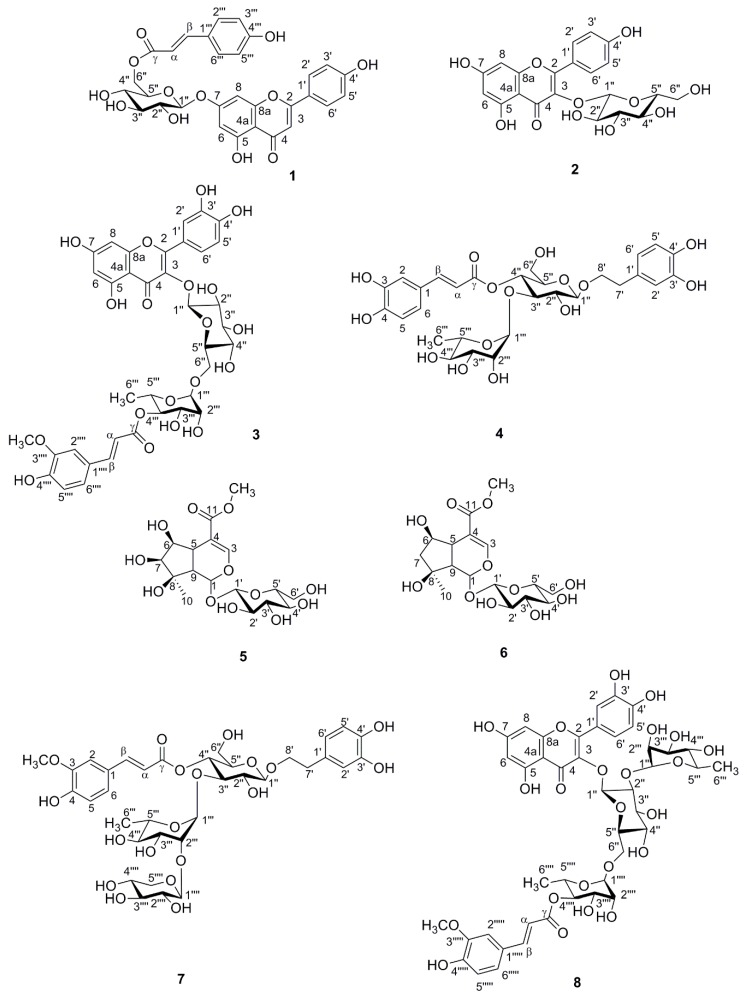
Structures of isolated compounds.

**Figure 2:**
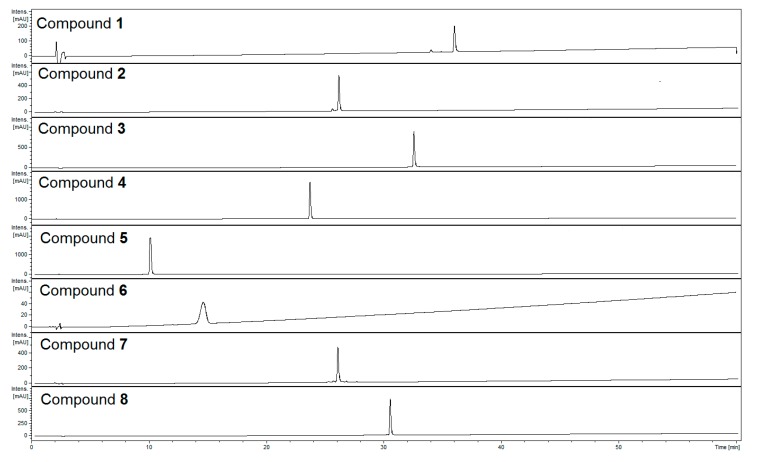
HPLC-chromatograms of isolated compounds recorded at λ = 240 nm. HPLC conditions: Zorbax SB-C_18_ (150 × 2.1 mm, 1.9 μm), mobile phase: A. 0.1% HCOOH/H_2_O; linear gradient 0–60 min, 5% to 60% B. 0.1% HCOOH/MeCN.

Phenylpropanoid glycosides, iridoids, and flavonoids represent the most abundant classes of compounds in the aqueous-methanolic extract of *Lamii albi herba*. Eight constituents of these classes (Figure 1) were isolated from the herb of *L. album*. Using ultra-high-performance liquid chromatography coupled with diode array detection and mass spectrometry (UHPLC-DAD-MS/MS) we determined retention times and confirmed the purity (Figure 2) as well as the molecular masses of the isolated compounds (Table 1).

Compounds were analyzed in both negative and positive ion modes. MS^2^ fragmentation was obtained for the two most abundant ions at the time. The detection of neutral loses was set for the moieties characteristic of glycoside fragmentation, such as at 162, 132, 176 assigned to hexose or caffeoyl residues, pentose, and uronic acid or feruloyl residues, respectively. In case of detection of one of the neutral loss masses, MS^3^ fragmentation was performed in order to obtain the fragmentation spectrum of the aglycone moiety. The main ion in the MS spectrum of verbascoside (**4**) (R_t_ = 23.9 min) was [M − H]^−^ (*m*/*z* 623) in the negative ESI mode, whereas the main ions in the MS^2^ pattern in negative ESI mode were at *m*/*z* 461 and *m*/*z* 315. The typical ion *m*/*z* 461 was detected in phenylpropanoids such as verbascoside and echinacoside, fragmentation was due to the loss of a caffeoyl unit (∆*m* = 162) in the MS^2^ pattern in negative ESI mode. Subsequent loss of rhamnose residue (∆*m* = 146) provides the ion at *m*/*z* 315 in negative ionization mode [15]. Many phenylpropanoids isolated previously from aerial parts of white dead nettle displayed similar verbascoside fragment ions in the negative ESI mode [12]. Herein, we have observed such similarities in the case of compound **7** (R_t_ = 26.1 min), which allowed us to classify it as a phenylpropanoid. The main ion in the MS spectrum was [M − H]^−^ (*m*/*z* 769) in negative ESI mode, wheras the major MS^2^ ions in the negative ionization mode were [M − H − 132]^−^ (*m*/*z* 637), [M − H − 162]^−^ (*m*/*z* 607), and [M − H − 176]^−^ (*m*/*z* 593) corresponding to the neutral losses of pentose, caffeoyl, and feruloyl residues, respectively. Compound **7** was finally identified as phlinoside D by comparing its ^1^H and ^13^C NMR spectra with the spectral data of phlinosides available in the literature [16,17,18]. The major ion in MS spectrum of compound **3** (R_t_ = 32.8 min) was [M − H]^−^ (*m*/*z* 785), and the MS^2^ fragmentation pattern showed signals at *m*/*z* 623 [M − H − 162]^−^, *m*/*z* 609 [M − H − 176]^−^, and *m*/*z* 591 [M − H − 194]^−^ due to the neutral losses of caffeoyl, feruloyl, and ferulic acid residues, respectively, in the negative ionization mode. These ions have been previously assigned to phenylpropanoid-like compounds. However, the MS^2^ fragmentation pattern of compound **3** as well as **8** (R_t_ = 30.7 min) showed signals at *m*/*z* 301 in the negative ionization mode, which is likely to be the ion of quercetin. The main ion of compound 8 in the MS spectrum was [M − H]^−^ (*m*/*z* 931), and fragmentary ions showed signals at *m*/*z* 785 [M − H − 146]^−^, *m*/*z* 609 [M − H − 146 − 176]^−^, and *m*/*z* 301 [M − H − 146 − 176 − 162 − 146]^−^. All MS data were compared with previous reports of similar structures [19]. In addition, the in-depth analysis of NMR spectra, including ^1^H, ^13^C, HSQC, and HMBC, and their comparison with the available literature on the reported isomers, allowed the structures of quercetin oligosaccharides to be established [19]. The structure of compound **3** was elucidated as quercetin 3-*O*-(4′′′-*O*-*E*-feruloyl)-*α*-rhamnopyranosyl-(1→6)]-*β*-glucopyranoside. The structure of compound **8** was quercetin 3-*O*-*α*-rhamnopyranosyl-(1→2)[(4′′′′-*O*-*E*-feruloyl)-*α*-rhamnopyranosyl-(1→6)]-*β*-glucopyranoside. Compounds **3** and **8** were identified as new compounds, which have not been described to date. The detailed ^1^H and ^13^C spectral data of compounds **3** and **8** have been provided in Table 2. UV–Vis absorption spectra allowed us to assign compounds **5** (R_t_ = 10.1 min) and **6** (R_t_ = 14.8 min) to the class of iridoids. In addition, the compounds **5** and **6** were characterized by [M + HCOOH − H]^−^ ions at *m*/*z* 467 and 451 in the negative ionization mode, respectively. The major ions from the MS^2^ fragmentation pattern were [M − H]^−^ (*m*/*z* 421) and [M − H]^−^ (*m*/*z* 405), respectively. In addition, the fragmentary peak *m*/*z* 259 of compound **5** was observed due to the direct loss of anhydroglucose (Δ*m* = 162). On the other hand, fragmentary ions at *m*/*z* 283 [M − 122]^−^ and *m*/*z* 225 [M − 162 − 18]^−^ of compound **6** were formed due to the loss of the 4-carbonylethylen piran ring and anhydroglucose along with dehydration [20]. Other fragmentation patterns of compound **5** with ions at *m*/*z* 867 and *m*/*z* 445 as well as compound 6 at *m*/*z* 835, *m*/*z* 445, and *m*/*z* 429 in the positive ESI mode have been also noted and compared with the available literature [21]. Compound **5** was identified as lamalbid, also named lamiridoside, whereas compound **6** was shanzhiside methyl ester.

In addition to MS data, the structures of compounds were confirmed by comparing their ^1^H NMR spectra with the spectral data available in the literature.

### 2.2. Cytotoxicity of Compounds

**Figure 3:**
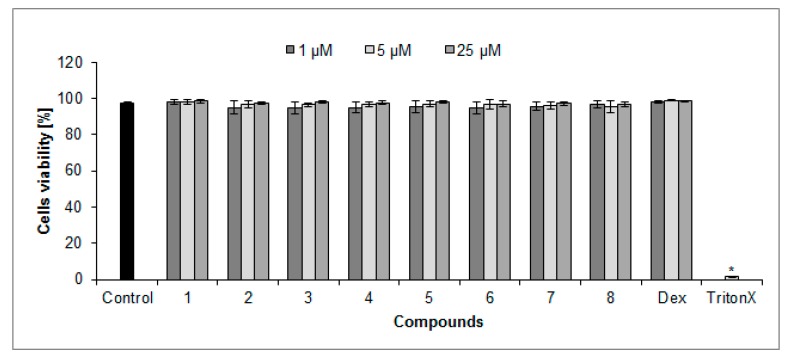
The polymorphonuclear leukocytes (PMNs) viability (%) upon 24 h-treatment with isolated compounds **1**–**8** and dexamethasone (Dex). Statistical significance was established by ANOVA with Tukey’s post hoc test. * *p* < 0.001 vs. control cells.

The isolated compounds in the concentration range of 1 to 25 µM did not influence PMNs viability (Figure 3). The population of propidium iodide (PI) positive cells in the nontreated control was 5.3 ± 1.5% after 24-h incubation. The population of PI positive cells ranged from 1.5 ± 0.2% to 3.4 ± 0.5% when cells were treated with compounds at the concentration of 25 µM. For comparison, the Dex-treated population of PI positive cells was 1.3 ± 0.1%, whereas TritonX, which was used as a positive control in cytotoxicity studies, displayed 98.5 ± 1.1% PI positive cells.

### 2.3. Inhibition of IL-8 Secretion

**Figure 4:**
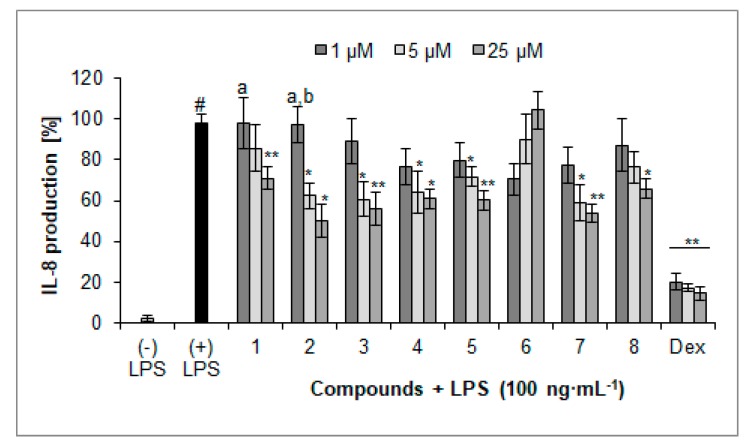
Inhibition of IL-8 production (%) by isolated compounds **1**–**8** in LPS-treated PMNs (mean ± S.E.M.). Dex—dexamethasone. Statistical significance of differences was established by ANOVA with Tukey’s post hoc test. * *p* < 0.05, ** *p* < 0.001 vs. (+) LPS; ^#^ *p* < 0.001 vs. (−) LPS; ^a^ *p* < 0.05 vs. a concentration of 25 µM; ^b^ *p* < 0.05 vs. a concentration of 5 µM.

Most of the compounds isolated from the herb of *L. album* were active inhibitors of inflammatory mediator secretion, in particular of chemokine secretion, such as IL-8 (Figure 4). All compounds, except for compound **6**, at the concentration of 25 µM significantly inhibited secretion of IL-8. The lowest values of released IL-8 were observed when cells were treated with compounds **2** (49.9 ± 8.1%), **7** (53.7 ± 4.5%), and **3** (56.0 ± 8.1%) at the concentration of 25 µM compared with nontreated control cells (97.6 ± 5.0%). On the other hand, a score of IL-8 release by cells treated with verbascoside (**4**) and compound **8** was less relevant 60.8 ± 5.1% and 65.9 ± 4.9% of the control, respectively. The identified iridoid (**5**) expressed activity similar to verbascoside (**4**), 60.1 ± 5.1% of control. Compound **1** showed less relevant activity (70.9 ± 5.5% compared to LPS-treated cells) at the highest concentration. It is worth to note that compounds **1** and **2** displayed concentration-dependent activity. However, none of these compounds reached activity comparable with the positive control, dexamethasone (14.7 ± 3.3%, 25 µM).

### 2.4. Inhibition of TNF-α Production

**Figure 5:**
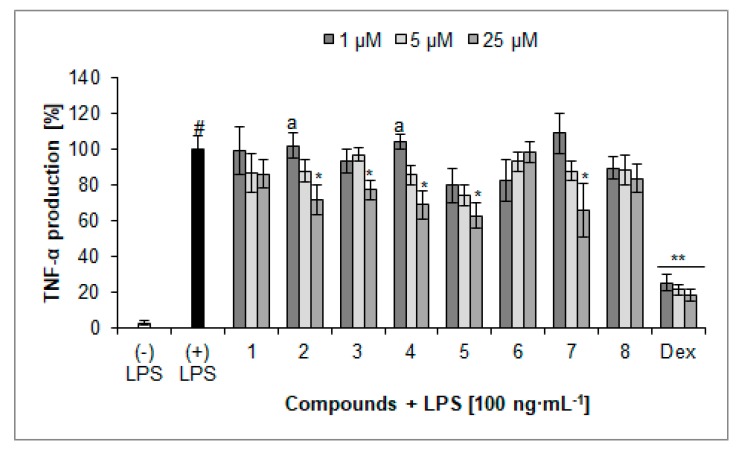
Inhibition of TNF-α production (%) by isolated compounds **1**–**8** in LPS-treated PMNs (mean ± S.E.M.). Dex—dexamethasone. Statistical significance of differences was established by ANOVA with Tukey’s post hoc test. * *p* < 0.05, ** *p* < 0.001 vs. (+) LPS; ^#^ *p* < 0.001 vs. (−) LPS; ^a^ *p* < 0.05 vs. a concentration of 25 µM.

Compounds **2**, **3**, **4**, **5**, and **7** at the concentration of 25 µM significantly inhibited the secretion of cytokine TNF-α (Figure 5). The cells treated with lamalbid (**5**) and phlinoside D (**7**) at the concentration of 25 µM released 62.8 ± 7.2% and 66.0 ± 14.6% of TNF-α, respectively, compared to control cells (100.0 ± 7.3%). In addition, astragalin (**2**) and verbascoside (**4**) treated cells produced 71.8 ± 8.4% and 68.9 ± 7.6% of TNF-α, respectively, as well as the concentration-dependent activity observed in the case of astragalin (**2**) and verbascoside (**4**). The percentage of TNF-α secretion by cells treated with LPS and dexamethasone as the positive control was 18.5 ± 3.7% compared to LPS-treated cells.

### 2.5. Inhibition of ROS Production

All tested compounds, except for astragalin (**2**), at the concentration of 25 µM, significantly inhibited the oxidative burst in stimulated PMNs (Table 3). In this assay, the most relevant inhibitors were derivatives of quercetin (compounds **3** and **8**). The cells treated with compounds **3** and **8** generated 16.9 ± 1.2% and 10.0 ± 2.6% ROS, respectively, compared to control cells (104.5 ± 8.3%). These results were similar to the results obtained for quercetin (8.8 ± 1.8%) used as a positive control. Phlinoside D (21.0 ± 3.4% compared to f-MLP-treated cells) inhibited ROS production even more significantly than verbascoside (42.6 ± 4.2% compared to f-MLP treated cells). The percentage of ROS production by cells treated by iridoids such as lamalbid (**5**) and shanzhiside methyl ester (**6**) as well as the apigenin derivative (**1**) was 68.6 ± 5.2%, 45.7 ± 1.4%, and 42.6 ± 5.1%, respectively, compared to f-MLP-treated cells.

## 3. Discussion

In the presented study, the role of compounds isolated from extract of *L. album* herb in the downregulation of inflammatory response of human neutrophils was determined. Although some of them, particularly apigenin 7-*O*-(*p*-coumaroyl)-glucoside (**1**), lamalbid (**5**), and phlinoside D (**7**), have been isolated previously; their biological activity has never been established according to our knowledge. Moreover, two new constituents of *L. album* herb, such as feruloyl glycosides of quercetin (**3** and **8**), have been isolated for the first time from the plant material, despite that their isomers are already known [19]. It is worth noting that extracts from the flowers or herb of white dead nettle were used in the treatment of inflammatory disorders of the skin and genital tract in folk medicine. Additionally, in some regions of Europe, they are still consumed in the everyday diet. Thus, searching the biological activity of white dead nettle’s constituents seems to be justified, particularly in order to support the dietary application of anti-inflammatory phytochemicals, which might lead to the limitation of noninfectious inflammation associated with chronic inflammatory diseases.

Neutrophils, through the generation of oxidative burst and release of other antimicrobial substances, constitute the first line of defense against pathogens [22]. Nevertheless, the role of overstimulated human neutrophils accompanied with prolonged reactive oxygen species (ROS) production in chronic inflammatory diseases, such as atherosclerosis, is widely considered [23,24]. Bearing in mind that inflammatory factors may trigger different signaling pathways, stimulation of cytokine production by lipopolysaccharide (LPS) was used. Reactive oxygen species play a crucial role in the progression of many inflammatory diseases acting both as a signaling molecule and as a mediator of inflammation. On the other hand, LPS signal transduction via Toll-like receptor 4 (TLR-4) initiates the production of cytokines [25]. However, there is more and more evidence that neutrophil-derived ROS enhance the production of pro-inflammatory cytokines, such as IL-1β and TNF-α, on LPS challenge [26] as well as intermediate LPS/TLR4-mediated IL-8 signaling [27]. Furthermore, some pro-inflammatory cytokines, such as TNF-α and IL-1β, are inducers of IL-8 production [28,29]. Antioxidant treatment decreased LPS-induced nuclear translocation of NF-κB as well as IL-8 production in human monocytes [24]. The antioxidant properties of natural compounds, including flavonoids, phenylpropanoids, and iridoids, have been widely proved to date [30,31,32]. On the other hand, the assessment of their effect on different inflammatory mediators allowed us to obtain more detailed report on potential anti-inflammatory activity of these so far not widely studied compounds.

One of the most relevant known phenylpropanoid glycosides is verbascoside (acteoside). In vitro and in vivo studies, including the intestinal inflammation model, demonstrating the antioxidant and anti-inflammatory activities of verbascoside have been previously reported. It was suggested that verbascoside shows direct and indirect antioxidant activity. Additionally, it decreases NOS activity and reduces NF-ĸB activation and nuclear translocation leading to the decrease of concentration of pro-inflammatory mediators [33,34]. Verbascoside has been previously shown not to inhibit H_2_O_2_-nontreatement IL-8 secretion, but it was able to inhibit IL-8 secretion as well as NF-ĸB translocation in human colon adenocarcinoma in conditions of oxidative stress [35]. It is worth noting that verbascoside, along with another phenylpropanoid glycoside, echinacoside, are considered phenolic compounds of olive fruits characterized as having an important role in human health and well-being [36]. However, in our study verbascoside exerted rather average activity compared to other phenylpropanoids, such as phlinoside D, isolated for the first time from the herb of *L. album.* In contrary to verbascoside, phlinoside D is characterized by the presence of feruloyl moiety instead of caffeoyl residue. We have previously established that *trans*-lamiuside E, which also possess two methoxyl groups in its structure, significantly inhibited IL-8 secretion. On the other hand, we made a conclusion that methoxy-phenylpropanoids might exert less relevant antioxidant activity [12]. In the current study, phlinoside D turned out to be one of the most significant inhibitors of IL-8 secretion and ROS production. It is believed that the reduction of NF-ĸB activation is a consequence of a decrease in extracellular **∙**O_2_^−^ production [33]. Therefore, the role of the phenylpropanoid constituents of *L. album* in the prevention of chronic diseases, along with its potential dietary supplementation, should not be neglected. On the other hand, the compounds, which were isolated for the first time and identified as quercetin feruloyl-glycosides, were compounds **3** and **8**. In our study, it was shown that quercetin derivatives attenuated the secretion of inflammatory mediators, in particular IL-8, in PMNs. Additionally, they were the most active antioxidants of all tested compounds. The feruloyl moiety in the structures of these compounds, as well as phlinoside D, might contribute to their activity. Furthermore, the presence of quercetin core may enhance the antioxidant properties of compounds **3** and **8** as well as regulate IL-8 secretion in this pathway.

Many bioactivities, including antioxidant, anti-inflammatory, immunomodulating, and antimicrobial, were assigned to the iridoids [32,37,38,39]. Recently, it was shown that lamalbid, at a concentration of 125 µg·mL^−1^ (corresponding to 296 µM), inhibited ROS generation by 57%, but did not affect iNOS and NF-ĸB activity [40]. In our study iridoids, including lamalbid, were tested at a lower concentration (25 µM) than in previous reports. For this reason it is difficult to refer to the previous results.

Last but not least, the activity of identified iridoids should not be neglected. Due to their different biosynthetic pathway their structures are not closely related with phenylpropanoids or flavonoids. Hydroxyl groups linked with a cyclopentan ring are found in their structures. However, we are not able to point out the direct structure–activity relationship of iridoids. Their antioxidant activity does not seem to affect cytokine secretion as it was noted in the case of shaznhiside methyl ester (**6**). On the other hand, their contribution is likely to support the activity of other constituents of *L. album* in term of its properties.

## 4. Materials and Methods

### 4.1. Chemicals

Acetonitrile (MeCN) of gradient grade for UHPLC, butanol (BuOH), chloroform (CHCl_3_), diethyl ether (DE), ethyl acetate (EA), ethanol, and methanol (MeOH) for extraction were obtained from POCH. HEPES, l-glutamine, luminol, and formyl-met-leu-phenylalanine were purchased from Sigma-Aldrich (Sigma-Aldrich, St. Louis, MO, USA). Lipopolysaccharide (LPS from *Escherichia coli* 0111:B4) was purchased from Merck (Merck, Kenilworth, NJ, USA). Human ELISA sets (IL-8 and TNF-α) and propidium iodide were obtained from BD Biosciences. Phosphate buffered saline (PBS) was purchased from Gibco (Gibco, HK, China). Penicillin–streptomycin were obtained from PAA. Hanks’ balanced salt solution (HBSS), RPMI 1640 medium, and fetal bovine serum (FBS), as well as formic acid eluent additive for UHPLC-MS, were purchased from Sigma-Aldrich (Sigma-Aldrich, St. Louis, MO, USA). Water was obtained using water purification system MILLIPORE Simfilter Simplicity UV.

### 4.2. Plant Material

*Lamium album* L. aerial parts were collected in May 2014 in Warsaw (52°12′47″ N, 20°59′52″ E). A specimen (No FW25_20140425_LA) of aerial parts is available in the herbarium of the Department of Pharmacognosy and Molecular Basis of Phytotherapy, Medical University of Warsaw. The plant material (Figure S1) was identified by Monika Czerwińska according to the guidebook [41,42].

### 4.3. Compounds Isolation

Isolation of compounds: 1370 g of powdered herb of *L. album* were macerated for 12 h and then exhaustively extracted three times with aqueous methanol (70%, *v*/*v*) (ratio 1:15 plant material: solvent; 30 °C, 3 h) in an ultrasonic bath. The organic solvent was evaporated under reduced pressure at 40 °C. Next, the aqueous methanol residue was partitioned between chloroform (5 × 1 L), diethyl ether (3 × 1 L), ethyl acetate (5 × 1 L), and *n*-butanol saturated with water (5 × 1 L), every time in ratio 1:1 of residue and solvent. The obtained fractions, such as DE (2.5 g), EA (7.4 g), and BuOH (47.3 g) were evaporated to dryness under vacuum and fractionated as follows. DE and EA residue was fractionated over silica gel (0.045–0.075 mm; 4 cm × 16 cm) using a CHCl_3_ gradient in EtOAc (100–0%), and a CHCl_3_ gradient in MeOH (0–100%) as an eluent, respectively. BuOH residue was fractioned over DIAION (6 × 20 cm) using a MeOH gradient in water (20–100%) as an eluent. The obtained fractions were pooled into six (DE1-DE6), nine (EA1-EA9), and five (BuOH1-BuOH5) main fractions based on their TLC profile (silica gel F254; ethyl acetate:formic acid:water (80:8:12, *v*/*v*/*v*), derivatized with 1% Natural Product Reagent (Carl Roth, Carl Roth, Karlsruhe, Germany).

Fraction DE6 (640 mg) was subjected to preparative HPLC (Shimadzu LC10vp, Japan, Zorbax SB-C_18_—5 mm, 150 mm × 21.2 mm, Agilent (Agilent, Santa Clara, CA, USA), 280 nm, flow 20 mL·min^−1^, mobile phase: 0.1% HCOOH in water (A) and 0.1% HCOOH in acetonitrile (B); elution program: 0% B–40% B (0–60 min), 40% B–50% B (60–65 min). Fractions were collected based on UV–Vis chromatograms to give pure compounds **1** (t_r_ = 46.70–47.25 min, 4.9 mg).

Fraction EA6 (1.2 g) was subjected to preparative HPLC (instrumentation and gradient elution as described above). Fractions were collected based on UV–Vis chromatograms to give pure compounds **2** (t_r_ = 33.30–33.70 min, 15.8 mg) and **3** (t_r_ = 42.80–43.20 min, 12.0 mg). Fraction EA8 (1.1 g) was subjected to column chromatography on Sephadex LH-20 (2.5 cm × 130 cm) eluted with MeOH (70%, *v*/*v*) to give 144 fractions combined to 11 main fractions EA8_1_-EA8_11_ based on TLC profile (ethyl acetate:formic acid:acetic acid:water (100:11:11:26, *v*/*v*/*v*/*v*), derivatized with 1% Natural Product Reagent, Carl Roth). Fraction EA8_5_ (195.8 mg) was subjected to preparative HPLC (instrumentation and gradient elution as described above). Fractions were collected based on UV–Vis chromatograms to yield pure compound **4** (t_r_ = 29.70–31.00 min, 106.2 mg).

Fractions BuOH2 (4.0 g) and BuOH4 (9.4 g) were subjected to column chromatography on Sephadex LH-20 (2.5 cm × 130 cm), eluted with MeOH (70%, *v*/*v*), to give each 144 fractions combined to six (BuOH2_1_-BuOH2_6_) and seven (BuOH4_1_-BuOH4_7_) main fractions, respectively, based on TLC profile (ethyl acetate:formic acid:acetic acid:water (100:11:11:26, *v*/*v*/*v*/*v*), derivatized with 1% Natural Product Reagent, Carl Roth, Karlsruhe, Germany). Next, fraction BuOH2_1_ (530 mg) was subjected to preparative HPLC (instrumentation and gradient elution as described above). Fractions were collected based on UV–Vis chromatograms to yield pure compound **5** (t_r_ = 13.20–13.80 min, 81.6 mg). Fractions BuOH4_1_ (2.0 g), BuOH4_2_ (0.4 g), and BuOH4_3_ (0.9 g) were subjected to column chromatography on Sephadex LH-20 (2.5 cm × 130 cm) eluted with MeOH (50%, *v*/*v*). Finally, using preparative HPLC (instrumentation and gradient elution as described above) pure compounds were obtained as follows, from fraction BuOH4_1B_ (944 mg): **6** (t_r_ = 32.20–32.40 min, 12.5 mg), from fraction BuOH4_2C_ (1 g): compound **7** (t_r_ = 33.50–34.00 min, 38.8 mg), and from fraction BuOH4_3C_ (160 mg): compound **8** (t_r_ = 40.40–40.70 min, 4.6 mg).

Structures of the isolated compounds have been determined based on UV–Vis, MS, and ^1^H spectra. If needed, additional experiments, such as ^13^C, HSQC, and HMBC, as well as chromatographic experiments, were performed. The NMR spectra were acquired using Varian VNMRS 300 Oxford 300 MHz (for compounds **1**, **2**, **4**, **5**, **6**, and **7**) and Bruker AVANCE 500 MHz spectrometers (Bruker, Billerica, MA, USA) (for compounds **3** and **8**). 

### 4.4. Phytochemical Analysis by HPLC-DAD-MS^n^ Method

HPLC-DAD-MS^n^ analysis was performed on a UHPLC-3000 RS system (Dionex, Sunnyvale, CA, USA) with DAD detection and an AmaZon SL ion trap mass spectrometer with ESI interface (Bruker Daltonik GmbH). Separation was performed on a Zorbax SB-C_18_ column (150 × 2.1 mm, 1.9 μm, Agilent). The column temperature was 25 °C. For preliminary phytochemical analysis of extracts the mobile phase (A) was 0.1% HCOOH in water and the mobile phase (B) was 0.1% HCOOH in acetonitrile, as well as a linear gradient system was used: 0–60 min 5–60% B. The flow rate was 0.2 mL·min^−1^. The column was equilibrated for 10 min between injections. UV spectra were recorded over a range of 200 to 450 nm, chromatograms were acquired at 240, 280, 325, and 350 nm. The LC eluate was introduced directly into the ESI interface without splitting. The nebulizer pressure was 40 psi; dry gas flow 9 L·min^−1^; dry temperature 300 °C; and capillary voltage 4.5 kV. Analysis was carried out using scan from *m*/*z* 200 to 2,200. Compounds were analyzed in negative ion mode. The MS^2^ fragmentation was obtained for the most abundant ion at the time.

### 4.5. PMNs Isolation

Buffy coats were obtained from healthy adult volunteers (<35 years old) from the Warsaw Blood Donation Centre. Donors were recognized as healthy according to medical history and routine laboratory test. Donors declared that they were nonsmokers and were not taking medication. They were clinically confirmed to be healthy, and a routine laboratory test showed values within normal range. Neutrophils were isolated by dextran sedimentation and centrifugation in a Ficoll Hypaque gradient (1500 rpm, 4 °C). Erythrocytes were removed by hypotonic lysis. The purity of neutrophils preparation was >97%. Following isolation, the cells were suspended in an appropriate medium, such as RPMI 1640 medium and were maintained at 4 °C before use [43].

### 4.6. Cytotoxicity

Neutrophils cytotoxicity was also assessed by flow cytometry with the propidium iodide assay. After 24-h incubation of neutrophils (2 × 10^6^ in RPMI 1640 medium) with the tested compounds at concentration range from 1 to 25 µM they were centrifuged at 2000 rpm for 10 min at 4 °C. PMNs were washed twice with PBS and resuspended in 500 μL of PBS containing 5 μL of propidium iodide (PI, 50 μg·mL^−1^) and left for 15 min in the dark. The cells were analyzed by flow cytometry FACSCalibur (Becton Dickinson), and data from 10,000 events were recorded. Cells that displayed high permeability to propidium iodide were expressed as a percentage of PI (+) cells. The results of the viability were acquired according to an equation: 100% − PI (+) cells%. TritonX was used as a positive control.

### 4.7. TNF-α and IL-8 Production by PMNs

Neutrophils (2 × 10^6^) were cultured in a 24-well plate in RPMI 1640 medium with 10% FBS, 10 mM HEPES, and 2 mM l-glutamine for 24 h at 37 °C with 5% CO_2_ in the absence or presence of compounds at concentrations of 25 µM added 30 min before the stimulation with LPS (100 ng·mL^−1^; lipopolysaccharide). The TNF-α and IL-8 released into cell supernatants was measured by enzyme-linked immunosorbent assay (ELISA) following the indications of the manufacturer. The effect on TNF-α and IL-8 production was calculated as the percentage of released agent in comparison with stimulated control without tested extract. Dexamethasone (Dex) was used as a positive control.

### 4.8. Evaluation of ROS Production by Human Neutrophils

The ROS production by f-MLP (formyl-met-leu-phenylalanine)-stimulated neutrophils was determined using luminol-dependent chemiluminescence, respectively. Following isolation, cells were resuspended in HBSS. Cell suspension (3.5 × 10^5^) was incubated with 50 μL of compounds at concentrations 25 µM, and luminol (20 mM) in a 96-well plate. The ROS production was initiated by the addition of f-MLP (0.1 μg·mL^−1^) to obtain a total volume of 200 μL per well. Changes in chemiluminescence were measured over a 40 min period at intervals of 2 min using the microplate reader (BioTek, Winooski, VT, USA). Background chemiluminescence produced by nonstimulated cells was also determined. The percentage of inhibition was calculated in comparison to the control without tested extract in the maximum of luminescence. Quercetin was used as a positive control.

### 4.9. Statistical Analysis

The results were expressed as a means ± S.E.M. Statistical significance of differences between means was established by ANOVA with Tukey’s *post hoc* test. *P* values below 0.05 were considered statistically significant. All analyses were performed using Statistica 10 (StatSoft).

## 5. Conclusions

In conclusion, the screening of activity of compounds isolated from herb of *L. album* revealed that phenylpropanoid glycosides along with iridoids and flavonoids might be the valuable bioactive compounds present in this species. In particular, the feruloyl and caffeoyl moieties, as well as quercetin core in the described structures, are likely to affect the signaling pathways of the inflammatory response. Thus, the antioxidant activity of these compounds probably influence cytokines secretion to some extent. Our study allowed us to make the conclusion that *L. album* might be a valuable source of bioactive compounds and might provide the constituents required to limit noninfectious inflammation associated with diseases such as cardiovascular disorders and diabetes type 2. Additionally, the synergistic or additive activity of the constituents of this plant material should not be excluded. However, further investigation is required to support the traditional and nutritional significance of *L. album*.

## Figures and Tables

**Table 1:** The spectral data of compounds isolated from the herb of *L. album*.

Analyte No.	Compound	UV λ_max_ [nm]	[M − H]^−^ *m*/*z*	Fragmentary Ions
**1**	Apigenin 7-*O*-*β*-d-(6′′-trans-*p*-coumaroyl)-glucoside	269, 317	577	307, 269
**2**	Kaempferol 3-*O*-glucoside (astragalin)	235sh, 265, 342	447	327, 285, 255
**3**	Quercetin 3-*O*-[(4′′′-*O*-*E*-feruloyl)-α-rhamnopyranosyl-(1→6)]-*β*-glucopyranoside	251, 333	785	623, 609, 591, 477, 301, 271
**4**	Verbascoside	245, 329	623	461, 315
**5**	Lamalbid (lamiridoside)	241	467 *	421, 259
**6**	Shanzhiside methyl ester	221	451 *	405, 283, 225, 179, 143
**7**	Phlinoside D	216, 328	769	637, 607, 593, 461
**8**	Quercetin 3-*O*-α-rhamnopyranosyl-(1→2)[(4′′′′-*O*-*E*-feruloyl)-*α*-rhamnopyranosyl-(1→6)]-*β*-glucopyranoside	251, 332	931	887, 785, 769, 755, 738, 702, 609, 562, 490, 301

* [M + HCOOH − H]^−^.

**Table 2:** ^1^H and ^13^C spectral data of compounds **3** and **8** (CD_3_OD).

Quercetin	Compound 3		Compound 8	
	^1^H	^13^C	^1^H	^13^C
2	-	159.1	-	158.8
3	-	135.3	-	134.2
4	-	179.4	-	179.4
4a	-	105.6	-	105.8
5	-	163.1	-	163.1
6	6.19 (d, *J* = 2.0 Hz)	99.9	6.17 (d, *J* = 2.1 Hz)	99.7
7	-	166.0	-	165.6
8	6.33 (d, *J* = 2.0 Hz)	94.8	6.30 (d, *J* = 2.1 Hz)	94.6
8a	-	158.5	-	158.5
1′	-	123.2	-	123.3
2′	7.65 (d, *J* = 2.1 Hz)	117.5	7.58 (d, *J* = 2.1 Hz)	117.5
3′	-	146.0	-	146.1
4′	-	149.7	-	149.4
5′	6.92 (d, *J* = 8.4 Hz)	116.1	6.94 (d, *J* = 8.2 Hz)	116.1
6′	7.64 (dd, *J* = 8.4, 2.1 Hz)	123.4	7.56 (m)	123.5
**d** **-Glucose**				
1′′	5.31 (d, *J* = 7.7 Hz)	103.8	5.78 (d, *J* = 7.7 Hz)	100.1
2′′	3.50 (m)	75.7	3.70 (m)	79.6
3′′	3.45 (m)	78.1	3.58 (m)	79.1
4′′	3.39 (m)	71.1	3.49 (m)	71.1
5′′	3.40 (m)	76.9	3.37 (m)	76.6
6′′	3.85, 3.53 (m)	68.0	3.83, 3.55 (m)	67.6
**l** **-Rhamnose I**				
1′′′	4.61 (d, *J* = 1.6 Hz)	102.1	4.61 (d, *J* = 1.6 Hz)	101.7
2′′′	3.75 (dd, *J* = 3.4, 1.6 Hz)	72.2	3.75 (m)	72.2
3′′′	3.80 (dd, *J* = 9.8, 3.4 Hz)	70.3	3.73 (m)	72.2
4′′′	4.92 (t, *J* = 9.8 Hz)	75.2	4.88 (t, *J* = 9.8 Hz)	75.1
5′′′	3.66 (m)	67.7	3.61 (m)	67.6
6′′′	0.90 (d, *J* = 6.3 Hz)	17.6	0.81 (d, *J* = 6.3 Hz)	17.4
**l** **-Rhamnose II**				
1′′′′			5.24 (d, *J* = 1.6 Hz)	102.3
2′′′′			3.98 (dd, *J* = 3.4, 1.7 Hz)	72.3
3′′′′			3.80 (m)	70.3
4′′′′			3.29 (m)	74.0
5′′′′			3.95 (m)	69.8
6′′′′			0.86 (d, *J* = 6.3 Hz)	17.3
Feruloyl (*/#)				
*α*	6.26 (d, *J* = 15.9 Hz)	116.3	6.16 (d, *J* = 15.9 Hz)	116.3
*β*	7.56 (d, *J* = 15.9 Hz)	146.8	7.53 (d, *J* = 15.9 Hz)	146.8
*γ*	-	169.0	-	169.0
1′′′′/1′′′′′	-	129.9	-	129.0
2′′′′/2′′′′′	7.13 (d, *J* = 2.1 Hz)	115.0	7.14 (d, *J* = 2.1 Hz)	115.2
3′′′′/3′′′′′	-	147.8	-	147.9
4′′′′/4′′′′′	-	151.4	-	151.4
5′′′′/5′′′′′	6.98 (d, *J* = 8.4 Hz)	112.5	7.00 (d, *J* = 8.4 Hz)	112.6
6′′′′/6′′′′′	7.10 (dd, *J* = 8.4, 2.1 Hz)	122.9	7.12 (dd, *J* = 8.3, 2.1 Hz)	122.8
OCH_3_	3.90 (s)	56.3	3.92 (s)	56.4

* Carbon numbers of compound 3/# carbon numbers of compound **8**.

**Table 3:** Effect of compounds (25 µM) on f-MLP-stimulated ROS generation (mean ± S.E.M. (%)).

Analyte	ROS Production [%] ^†^
(−) f-MLP	31.3 ± 2.6
(+) f-MLP	104.5 ± 8.3 ^#^
1	42.6 ± 5.1 *
2	92.7 ± 6.2
3	16.9 ± 1.2 **
4	42.6 ± 4.2 *
5	68.6 ± 5.2 *
6	45.7 ± 1.4 *
7	21.0 ± 3.4 **
8	10.0 ± 2.6 **
Quercetin	8.8 ± 1.8 **

†% of ROS production compared to (+) f-MLP (100% production); * *p* < 0.05, ** *p* < 0.001 vs. stimulated control (control st.), ^#^ *p* < 0.05 vs. control n.st. (Not stimulated control).

## Supplementary Materials

The supplementary materials are available online. The image of plant material, the draft of isolation of compounds from aqueous-methanolic extract of *L. album* herb, and the ^1^H NMR spectra of the isolated compounds are available as Supplementary Materials.

## References

[1] Yalçin F.N., Kaya D. (2006). Ethnobotany, pharmacology and phytochemistry of the genus *Lamium* (Lamiaceae). Fabad J. Pharm. Sci..

[2] Turner N., Łuczaj Ł., Migliorini P., Pieroni A., Dreon A., Sacchetti L. (2011). Edible and tended wild plants, traditional ecological knowledge and agroecology. Crit. Rev. Plant Sci..

[3] Łuczaj Ł. (2008). Archival data on wild food plants used in Poland in 1948. J. Ethnobiol. Ethnomed..

[4] Heinrich M., Müller W., Galli C. (2006). Local Mediterranean Food Plants and Nutraceuticals.

[5] Pereira O.R., Domingues M.R.M., Silva A.M.S., Cardoso S.M. (2012). Phenolic constituents of *Lamium album*: Focus on isoscutellarein derivatives. Food Res. Int..

[6] Yordanova Z.P., Zhiponova M.K., Iakimova E.T., Dimitrova M.A., Kapchina-Toteva V.M. (2014). Revealing the reviving secret of the white dead nettle (*Lamium album* L.). Phytochem. Rev..

[7] Vogt T. (2010). Phenylpropanoid biosynthesis. Mol. Plant.

[8] Seigler D.S. (1998). Phenylpropanoids. Plant Secondary Metabolism.

[9] Kurkin V. (2003). Phenylpropanoids from medicinal plants: Distribution, classification, structural analysis, and biological activity. Chem. Nat. Comp..

[10] Bruneton J. (1999). Pharmacognosy: Phytochemistry of Medicinal Plants.

[11] Budzianowski J., Skrzypczak L. (1995). Phenylpropanoid esters from *Lamium album* flowers. Phytochemistry.

[12] Czerwińska M.E., Świerczewska A., Woźniak M., Kiss A.K. (2017). Bioassay-guided isolation of iridoids and phenylpropanoids from aerial parts of *Lamium album* and their anti-inflammatory activity in human neutrophils. Planta Med..

[13] Damtoft S., Jensen S.R., Nielsen B.J. (1991). Biosynthesis of iridoid glucosides in *Lamium album*. Phytochemistry.

[14] Alipieva K.I., Taskova R.M., Jensen S.R., Handjieva N.V. (2006). Iridoid glucosides from *Lamium album* and *Lamium maculatum* (Lamiaceae). Biochem. Syst. Ecol..

[15] Mitreski I., Stanoeva J.P., Stefova M., Stefkov G., Kulevanova S. (2014). Polyphenols in representative *Teucrium* species in the flora of R. Macedonia: LC/DAD/ESI-MS(n) profile and content. Nat. Prod. Commun..

[16] Calis I., Basaran A.A., Saracoglu I., Sticher O., Ruedi P. (1990). Phlinosides A, B and C, three phenylpropanoid glycosides from *Phlomis linearis*. Phytochemistry.

[17] Calis I., Basaran A.A., Saracoglu I., Sticher O., Ruedi P. (1991). Phlinosides D and E, phenylpropanoid glycosides, and iridoids from *Phlomis linearis*. Phytochemistry.

[18] Yalçin N.F., Ersöz T., Bedir E., Şahpaz S., Bailleul F., Khan I.A., Dönmez A.A., Çalis I. (2005). Phlinoside F, a new phenylethanoid glycoside from *Phlomis angustissima*. Turk. J. Chem..

[19] Veitch N.C., Regos I., Kite G.C., Treutter D. (2011). Acylated flavonol glycosides from the forage legume, *Onobrychis viciifolia* (sainfoin). Phytochemistry.

[20] Wu Q., Yuan Q., Liu E., Qi L., Bi Z., Li P. (2010). Fragmentation study of iridoid glycosides and phenylpropanoid glycosides in Radix Scrophulariae by rapid resolution liquid chromatography with diode-array detection and electrospray ionization time-of-flight mass spectrometry. Biomed. Chromatogr..

[21] Alipieva K., Kokubun T., Taskova R., Evstatieva L., Handjieva N. (2007). LC-ESI-MS analysis of iridoid glucosides in *Lamium* species. Biochem. Syst. Ecol..

[22] Quade M.J., Roth J.A. (1997). A rapid, direct assay to measure degranulation of bovine neutrophil primary granules. Vet. Immunol. Immunopathol..

[23] Baetta R., Corsini A. (2010). Role of polymorphonuclear neutrophils in atherosclerosis: Current state and future perspectives. Atherosclerosis.

[24] Mittal M., Siddiqui M.R., Tran K., Reddy S.P., Malik A.B. (2014). Reactive oxygen species in inflammation and tissue injury. Antioxid. Redox Signal..

[25] Palsson-McDermott E.M., O’Neill L.A. (2004). Signal transduction by the lipopolysaccharide receptor, Toll-like receptor-4. Immunology.

[26] Bulua A.C., Simon A., Maddipati R., Pelletier M., Park H., Kim K.Y., Sack M.N., Kastner D.L., Siegel R.M. (2011). Mitochondrial reactive oxygen species promote production of proinflammatory cytokines and are elevated in TNFR1-associated periodic syndrome (TRAPS). J. Exp. Med..

[27] Ryan K.A., Smith M.F., Sanders M.K., Ernst P.B. (2004). Reactive oxygen and nitrogen species differentially regulate Toll-like receptor 4-mediated activation of NF-kappa B and interleukin-8 expression. Infect. Immun..

[28] Bickel M. (1993). The role of interleukin-8 in inflammation and mechanisms of regulation. J. Periodontol..

[29] Harada A., Sekido N., Akahoshi T., Wada T., Mukaida N., Matsushima K. (1994). Essential involvement of interleukin-8 (IL-8) in acute inflammation. J. Leukoc. Biol..

[30] Panche A.N., Diwan A.D., Chandra S.R. (2016). Flavonoids: An overview. J. Nutr. Sci..

[31] Xue Z., Yang B. (2016). Phenylethanoid glycosides: Research advances in their phytochemistry, pharmacological activity and pharmacokinetics. Molecules.

[32] Tundis R., Loizzo M.R., Menichini F., Statti G.A., Menichini F. (2008). Biological and pharmacological activities of iridoids: Recent developments. Mini Rev. Med. Chem..

[33] Speranza L., Franceschelli S., Pesce M., Reale M., Menghini L., Vinciguerra I., De Lutiis M.A., Felaco M., Grilli A. (2010). Antiinflammatory effects in THP-1 cells treated with verbascoside. Phytother. Res..

[34] Hausmann M., Obermeier F., Paper D.H., Balan K., Dunger N., Menzel K., Falk W., Schoelmerich J., Herfarth H., Rogler G. (2007). In vivo treatment with the herbal phenylethanoid acteoside ameliorates intestinal inflammation in dextran sulphate sodium-induced colitis. Clin. Exp. Immunol..

[35] Ye Y.L., Chang H.S., Tseng Y.F., Shi L.S. (2017). Suppression of IL-8 release by sweet olive ethanolic extract and compounds in WiDr colon adenocarcinoma cells. J. Food Sci..

[36] Serreli G., Incani A., Atzeri A., Angioni A., Campus M., Cauli E., Zurru R., Deiana M. (2017). Antioxidant effect of natural table olives phenolic extract against oxidative stress and membrane damage in enterocyte-like cells. J. Food Sci..

[37] Kupeli E., Harput U.S., Varel M., Yesilada E., Saracoglu I. (2005). Bioassay-guided isolation of iridoid glucosides with antinociceptive and anti-inflammatory activities from *Veronica anagallis-aquatica* L.. J. Ethnopharmacol..

[38] Koo H.J., Lim K.H., Jung H.J., Park E.H. (2006). Anti-inflammatory evaluation of gardenia extract, geniposide and genipin. J. Ethnopharmacol..

[39] Gyurkovska V., Alipieva K., Maciuk A., Dimitrova P., Ivanovska N., Haas C., Bley T., Georgiev M. (2011). Anti-inflammatory activity of Devil’s claw in vitro systems and their active constituents. Food Chem..

[40] Mohamed N.M., Makboul M.A., Farag S.F., Tarawenh A.H., Khan S.I., Brooks T.A., Wang Y.-H., Roos S.A. (2017). Iridoid and phenylpropanoid glycosides from the roots of *Lantana montevidensis*. Med. Chem. Res..

[41] Tutin T., Heywood V., Burges N., Moore D., Valentine D., Walters S., Webb D. (1972). Flora Europaea.

[42] Rutkowski L. (2011). Klucz Do Oznaczania Roślin Naczyniowych Polski Niżowej.

[43] Böyum A. (1968). A one-stage procedure for isolation of granulocytes and lymphocytes from human blood. General sedimentation properties of white blood cells in a 1g gravity field. Scand. J. Clin. Lab. Invest..

