# Supplementary material for: Bioactive Constituents of Lamium album L. as Inhibitors of Cytokine Secretion in Human Neutrophils
Source: Molecules. 2018 Oct 25;23(11):2770. doi: 10.3390/molecules23112770 (PMC6278331; doi:10.3390/molecules23112770)
Supplement: Supplementary file 1 [file molecules-23-02770-s001.pdf]

Supplementary Materials

Bioactive constituents of *Lamium album* L. as inhibitors of cytokines secretion in human neutrophils

Monika E. Czerwińska [1,*], Anita Świerczewska [1], Sebastian Granica [1]

[1] Department of Pharmacognosy and Molecular Basis of Phytotherapy, Medical University of Warsaw, 1 Banacha street, 02-097 Warsaw, Poland

* Correspondence: mczerwinska@wum.edu.pl; Tel.: +48-22-572-0953

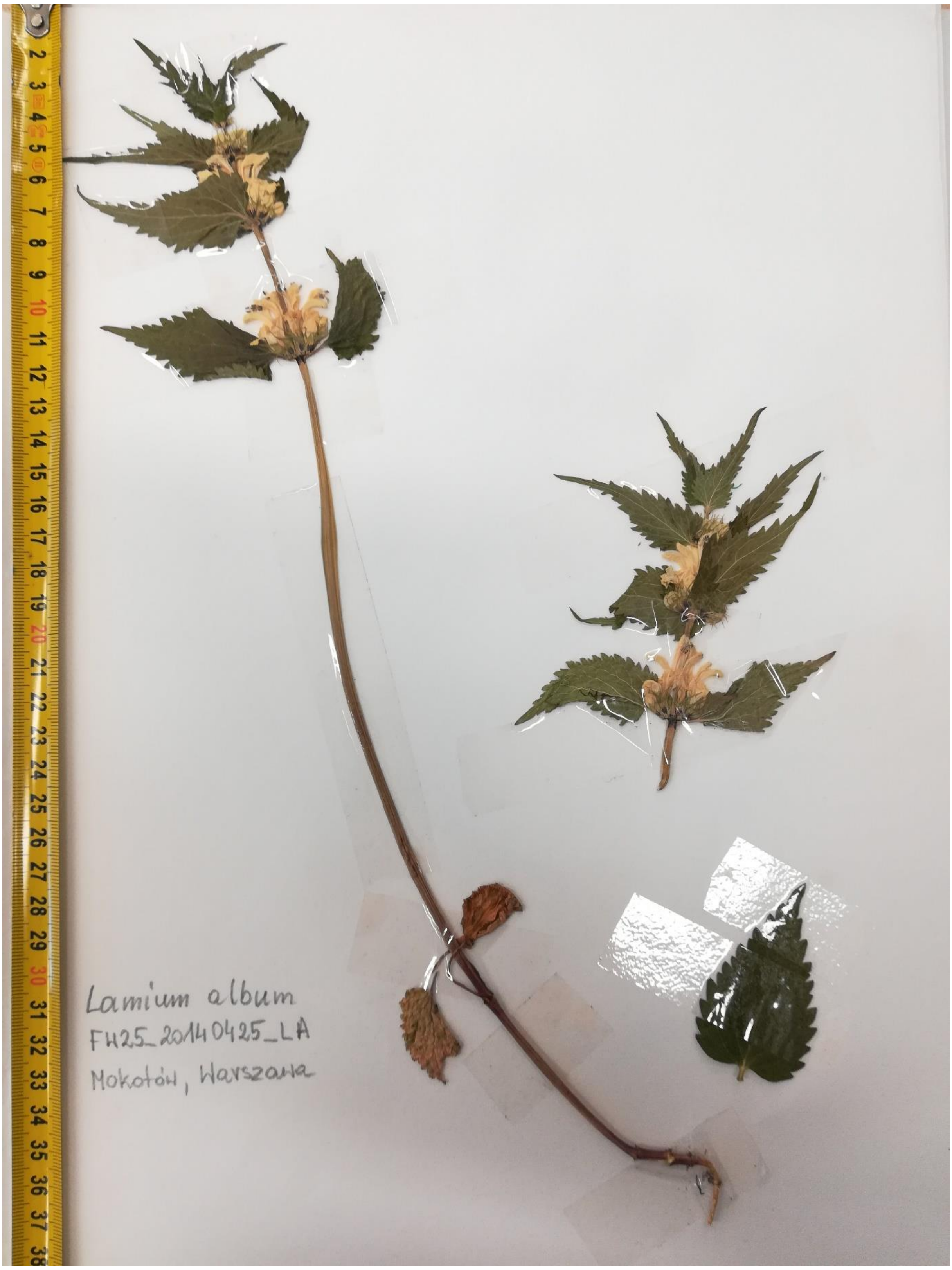

Figure S1. The voucher specimen of collected herb of *L. album* used for the isolation of compounds.

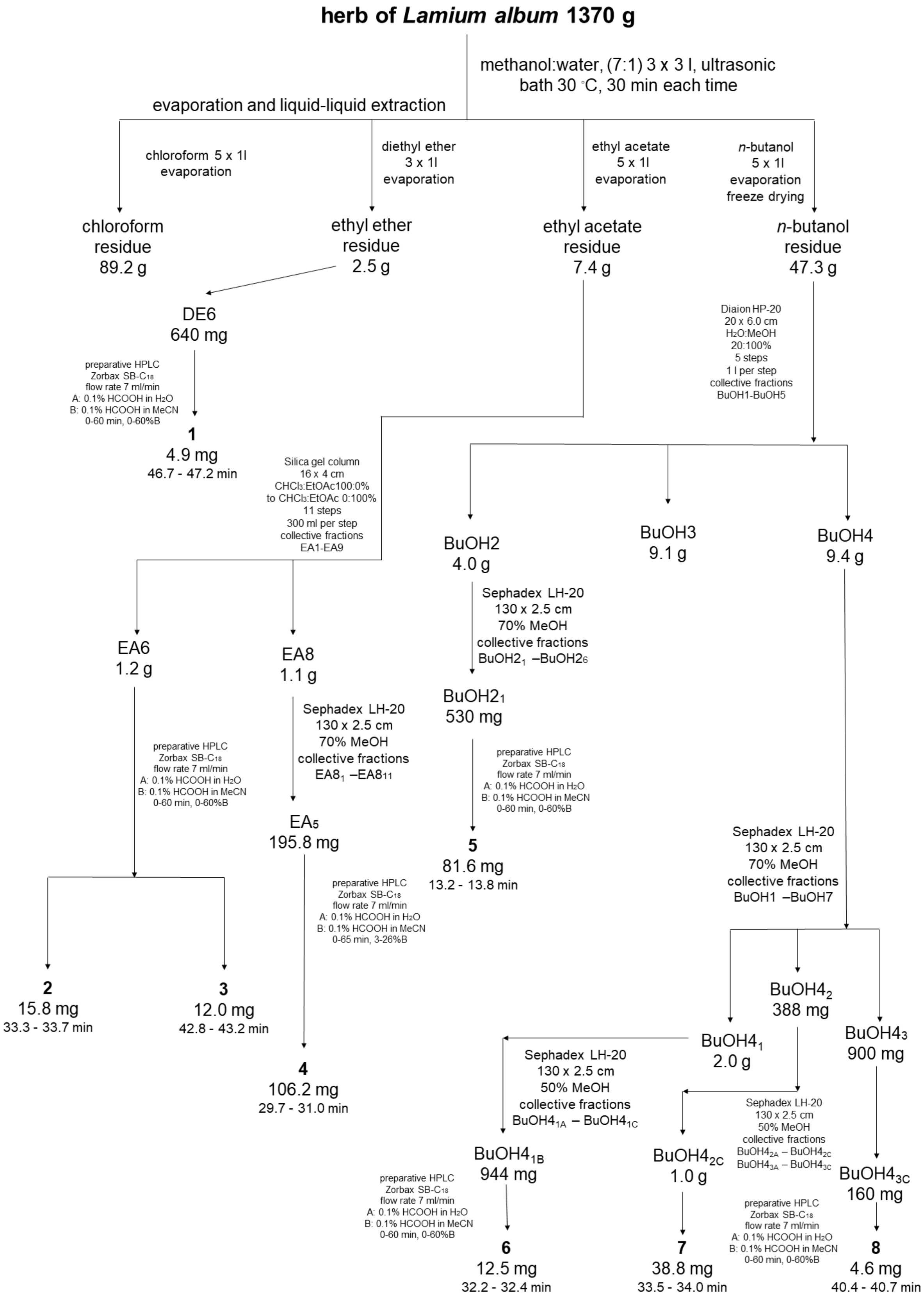

Figure S2. The draft of isolation of compounds from aqueous-methanolic extract from herb of *L. album*.

Compound **1**

Apigenin 7-*O*-*β*-D-(6''-trans-*p*-coumaroyl)-glucoside

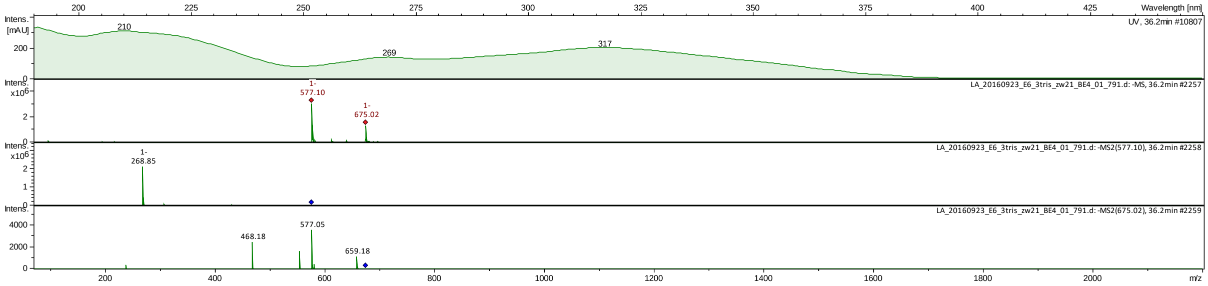

^{1}H NMR (300 MHz, DMSO) δ 12.97 (1H, s, 5-OH), 7.94 (2H, d, J = 8.8 Hz, H-2', H-6'), 7.51 (1H, s, , H-β), 7.36 (1H, d, J = 8.6 Hz, H-2''', H-6'''), 6.92 (2H, d, J = 8.7 Hz, H-3', H-5'), 6.82 (2H, d, J = 6.9 Hz, H-1, H-8), 6.67 (2H, d, J = 8.6 Hz, H-3''', H-5'''), 6.47 (1H, s, H-6), 6.30 (1H, s, H-*α*), 5.16 (1H, d, J = 7.0 Hz, H-1''), 4.46 (1H, d, J = 10.9 Hz, H-6''*α*), 4.21 – 4.09 (1H, m, H-6''β), 3.83 (1H, s, H-5'').

Compound **1** was identified by comparing the above spectral data with those in the literature [1, 2].

Compound **2**

Astragalin

Kaempferol 3-*O*-*β*-glucopyranoside

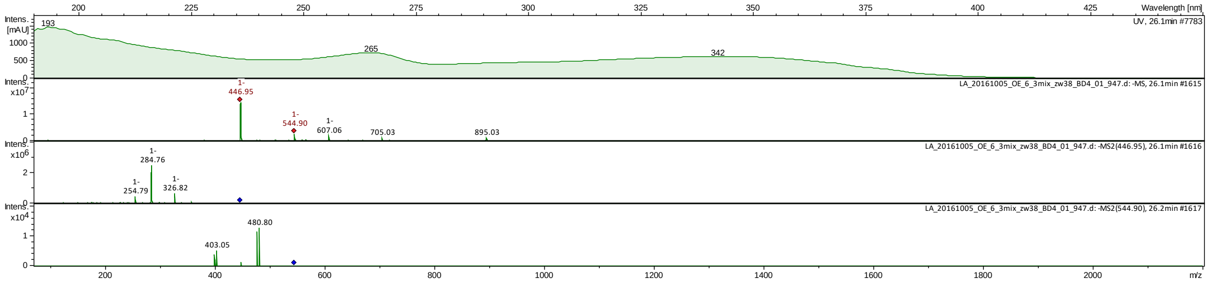

^{1}H NMR (300 MHz, CD_3OD) *δ*: 6.16 (1H, d, *J* = 1.8 Hz, H-6), 6.34 (1H, d, *J* = 1.8 Hz, H-8), 8.05 (2H, d, *J* = 8.3 Hz, H-2', 6'), 6.86 (2H, d, *J* = 8.3 Hz, H-3', 5'), 5.25 (1H, d, *J* = 6.1 Hz, H-1''), 3.73-3.20 (m). Compound **3** was identified by comparing the above spectral data with those in the literature [3].

Compound **3**

Quercetin 3-*O*-(4'''-*O*-*E*-feruloyl)-*α*-rhamnopyranosyl-(1→6)-*β*-glucopyranoside

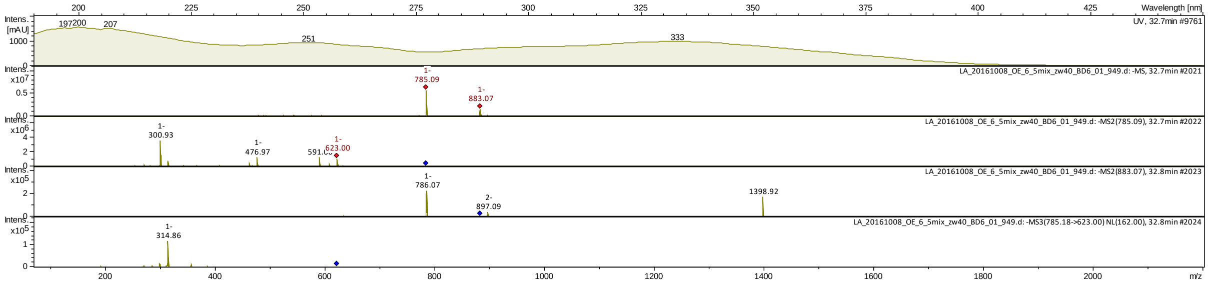

Compound **4**

Verbascoside

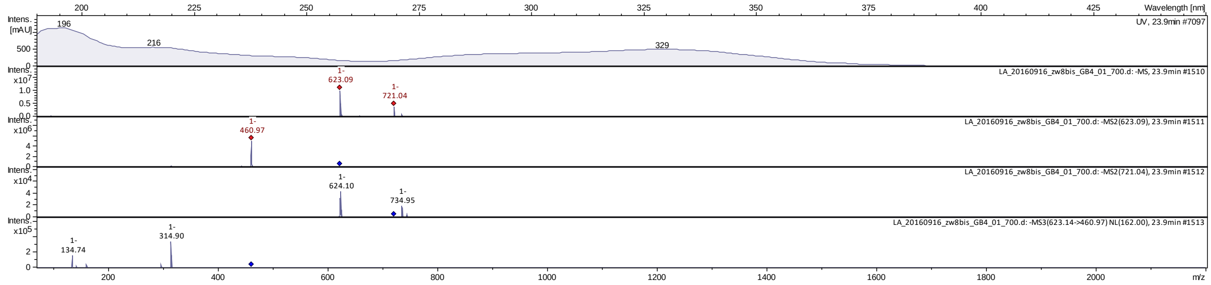

^{1}H NMR (300 MHz, CD_3OD) δ 7.59 (1H, d, J = 15.9 Hz, H-β), 7.05 (1H, d, J = 1.8 Hz, H-2), 6.96 (1H, dd, J = 8.3, 1.8 Hz, H-6), 6.78 (1H, d, J = 8.2 Hz, H-5), 6.70 (1H, d, J = 2.4 Hz, H-2'), 6.66 (1H, s, H-5'), 6.57 (1H, dd, J = 8.1, 1.9 Hz, H-6'), 6.27 (1H, d, J = 15.9 Hz, H-α), 5.19 (1H, s, H-1'''), 4.92 (1H, t, J = 9.4 Hz, H-4''), 4.38 (1H, d, J = 7.9 Hz, H-1''), 4.05 (1H, dd, J = 16.9, 7.4 Hz, H-8'α), 3.92 (1H, s, H-2'''), 3.83 (1H, d, J = 9.2 Hz, H-3''), 3.75 (1H, dd, J = 15.5, 7.8 Hz, H-8'β), 3.61 (1H, dd, J = 10.5, 6.3 Hz, H-6''α), 3.56 (2H, m, overlapped H-6''β, H-3'''), 3.52 (1H, s, H-5'''), 3.39 (1H, t, H-2''), 3.27 (1H, t, H-4'''), 2.80 (t, J = 7.4 Hz, H-7'), 1.09 (3H, d, J = 6.2 Hz, H-6'''). Compound **4** was identified by comparing the above spectral data with those in the literature [4].

Compound **5**

Lamalbid (lamiridoside)

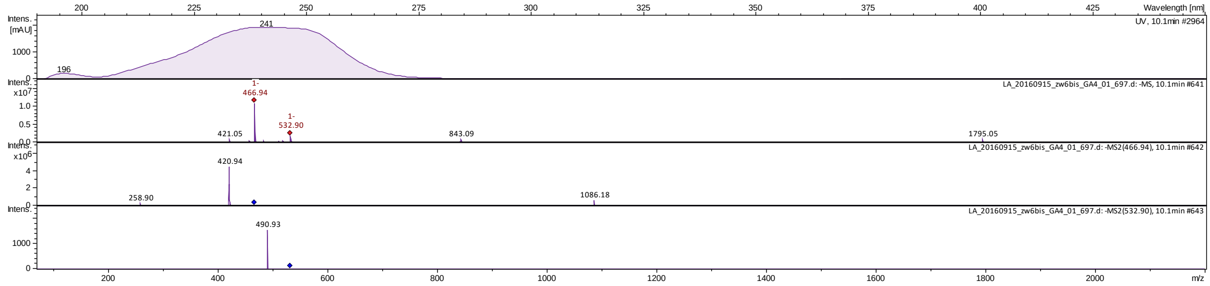

^{1}H NMR (300 MHz, CD_3OD) δ 7.40 (1H, s, H-3), 5.62 (1H, d, J = 1.7 Hz, H-1), 4.61 (1H, d, J = 7.9 Hz, H-1'), 3.95 (1H, t, J = 3.9 Hz, H-6), 3.89 (1H, dd, J = 11.9, 1.7 Hz, CH_2-6'), 3.73 (3H, s, OCH_3), 3.65 (1H, dd, J = 11.9, 5.7 Hz, CH_2-6'), 3.54 (1H, d, J = 4.4 Hz, H-7), 3.36 (1H, t, J = 6.9 Hz, H-3'), 3.30 (1H, m, H-5'), 3.24 (1H, d, J = 9.3 Hz, H-4'), 2.93 (1H, dd, J = 10.7, 2.6 Hz, H-5), 2.80 (1H, d, J = 12.0 Hz, H-9), 1.20 (3H, s, CH_3-10). Compound **5** was identified by comparing the above spectral data with those in the literature [5].

Compound **6**

Shanzhiside methyl ester

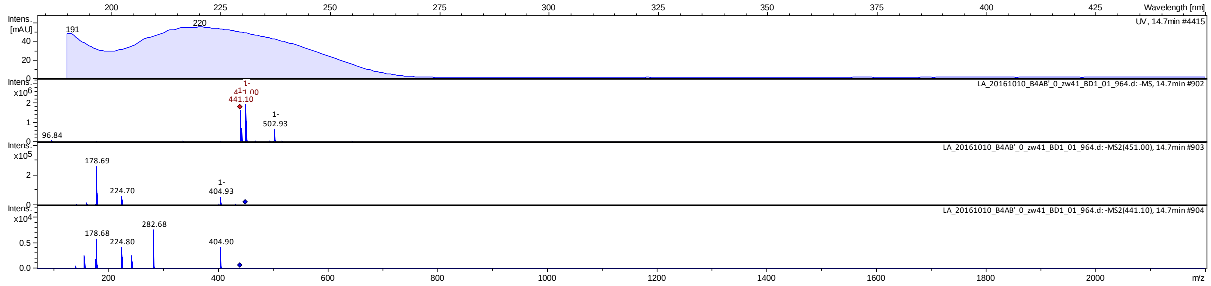

^{1}H NMR (300 MHz, CD_3OD) δ: 7.41 (1H, s, H-3), 5.61 (1H, d, *J* = 1.6 Hz, H-1α), 4.85 (1H, d, *J* = 8.0 Hz, H-1'), 3.74 (3H, s, OCH_3), 3.00 (1H, m, H-5β), 2.62 (1H, m, H-9β), 1.90 (2H, m, H-7), 1.25 (3H, s, CH_3-10). Compound **6** was identified by comparing the above spectral data with those in the literature [6]

Compound **7**

Phlinoside D

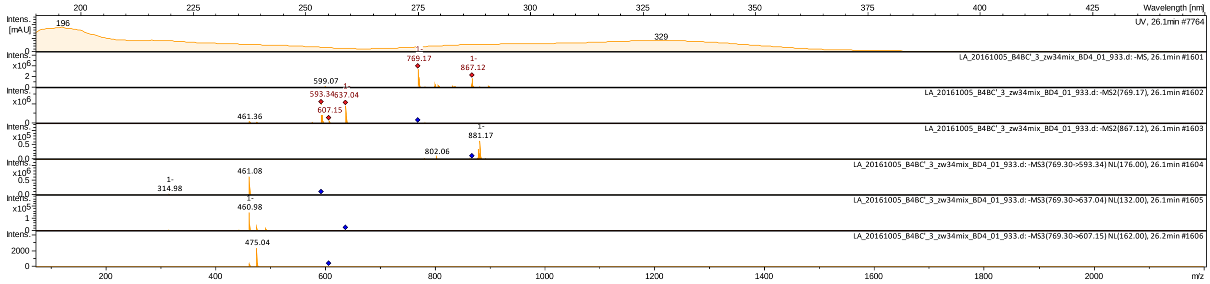

^{1}H NMR (300 MHz, CD_3OD) δ 7.66 (1H, d, J = 15.8 Hz, H-α), 7.20 (1H, s, H-2), 7.09 (1H, d, J = 8.2 Hz, H-6), 6.82 (1H, d, J = 8.2 Hz, H-5), 6.70 (1H, d, J = 1.9 Hz, H-5'), 6.68 (1H, d, J = 8.1 Hz, H-2'), 6.57 (1H, d, J = 8.1 Hz, H-6'), 6.38 (1H, d, J = 15.8 Hz, H-β), 5.49 (1H, s, H-1'''), 4.94 (1H, d, J = 8.8 Hz, H-4''), 4.38 (1H, d, J = 7.8 Hz, H-1''), 4.32 (1H, d, J = 7.1 Hz, H-1''''), 4.06 (1H, dd, J = 16.9, 7.5 Hz, H-8'), 3.95 (1H, s, H-2''''), 3.89 (3H, s, $-OCH_3$, H-2''', H-6''β), 3.78 (1H, t, H-3''), 3.56 (2H, d, J = 6.4 Hz, H-6''α, H-5'''), 3.52 (1H, s, H-5''''), 3.39 (1H, m, H-2''), 3.27 (1H, d, J = 9.6 Hz, H-4'''), 2.80 (2H, t, J = 7.3 Hz, H-7'), 1.10 (3H, dd, J = 17.7, 6.1 Hz, H-6''').

^{13}C NMR (75 MHz, CD_3OD) δ 168.22 (C-γ), 150.80 (C-4), 149.38 (C-3), 147.86 (C-β), 146.13 (C-3'), 144.67 (C-4'), 131.47 (C-1'), 127.65 (C-1), 124.34 (C-6), 121.25 (C-6'), 117.11 (C-5'), 116.49 (C-5), 116.28 (C-2'), 115.09 (C-α), 111.77 (C-2), 107.48 (C-1''''), 104.21 (C-1''), 101.96 (C-1'''), 82.82 (C-2'''), 82.27 (C-3''), 76.03 (C-2''), 74.37 (C-5''), 74.20 (C-4'''), 72.82 (C-2''''), 72.26 (C-8'), 71.94 (C-3'''), 70.53 (C-4''), 70.34 (C-5'''), 69.84 (C-4''''), 67.32 (C-5''''), 62.34 (C-6''), 56.45 ($-OCH_3$/C-3), 36.58 (C-7'), 18.41 (C-6'''). Compound **7** was identified by comparing the above spectral data with those in the literature [7-9].

Compound **8**

Quercetin 3-*O*-*α*-rhamnopyranosyl-(1→2)[(4'''-*O*-*E*-feruloyl)-*α*-rhamnopyranosyl-(1→6)]-*β*-glucopyranoside

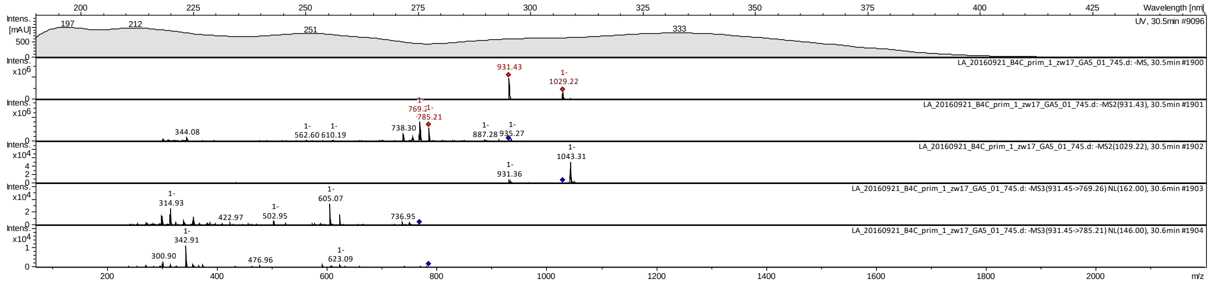

A

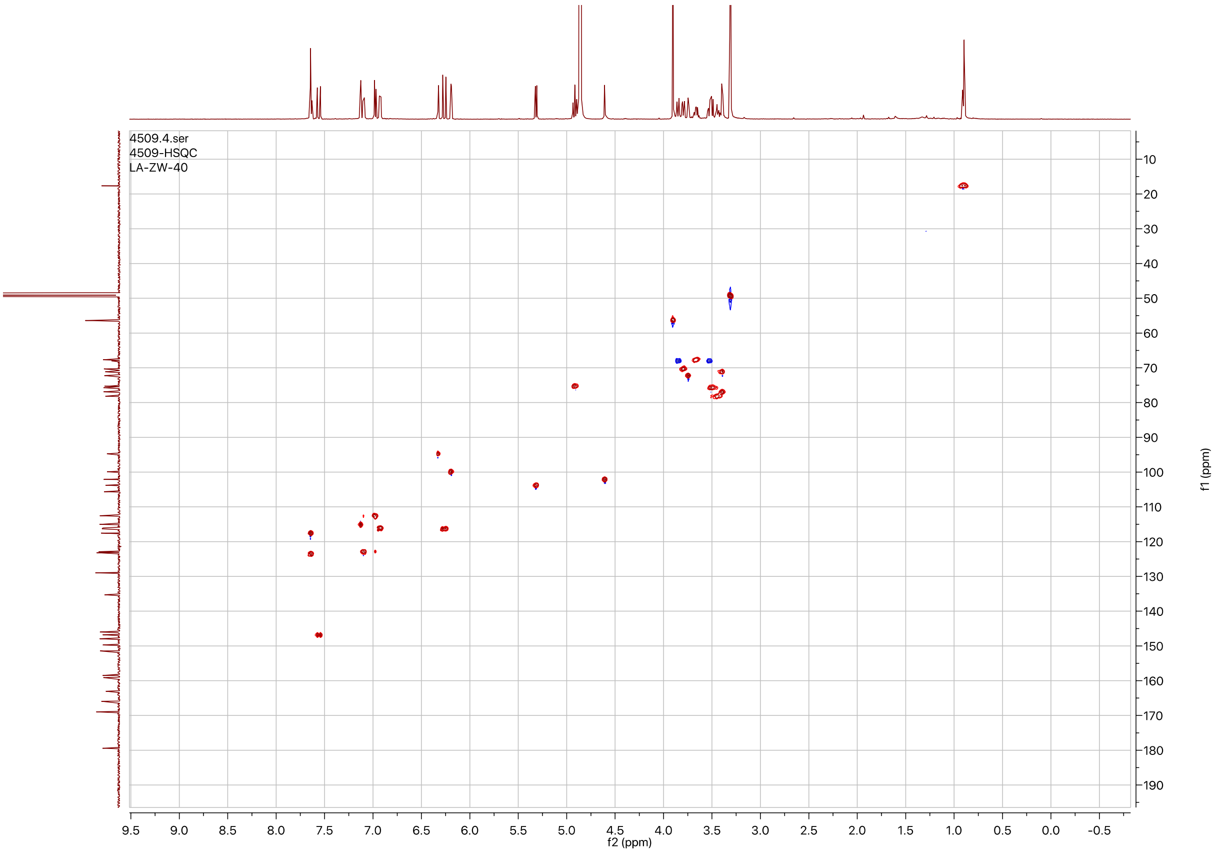

B

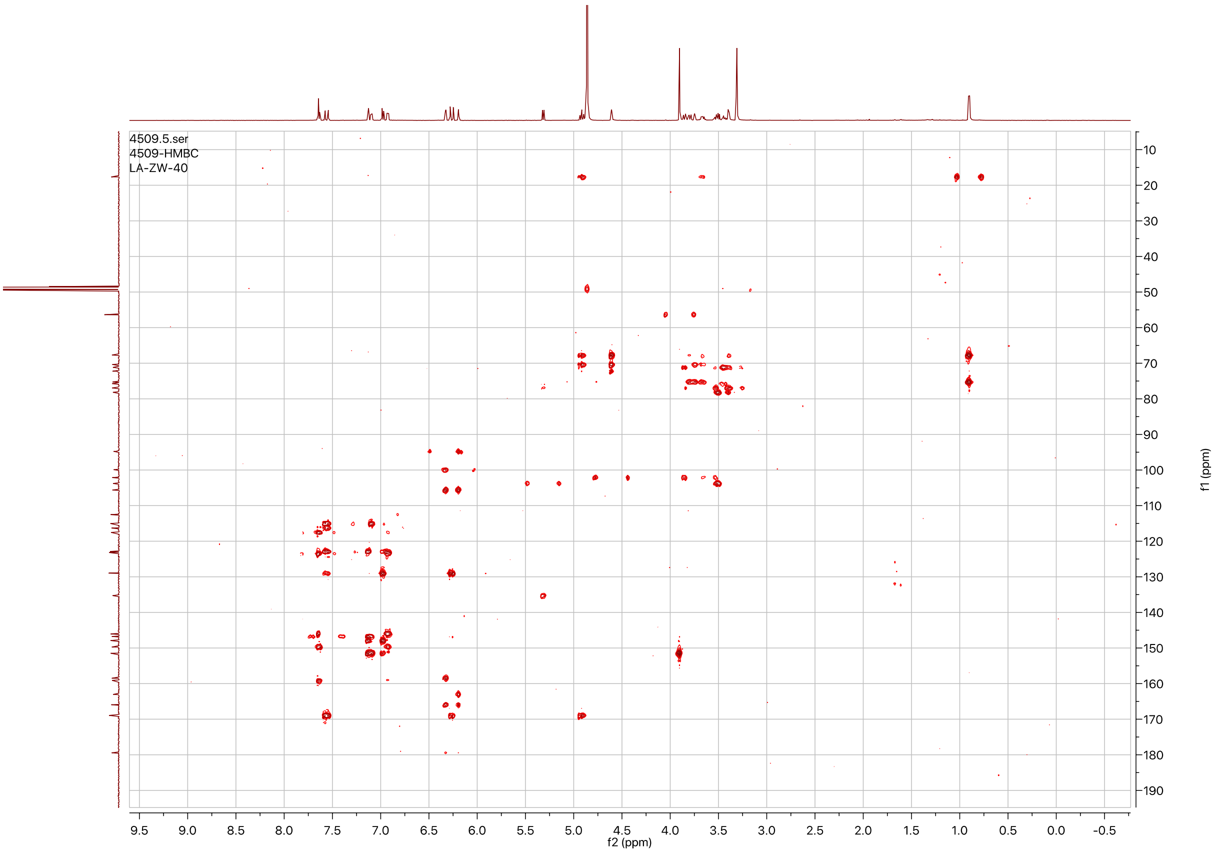

Figure S3. The HSQC (A) and HMBC (B) spectra of compound **3**.

A

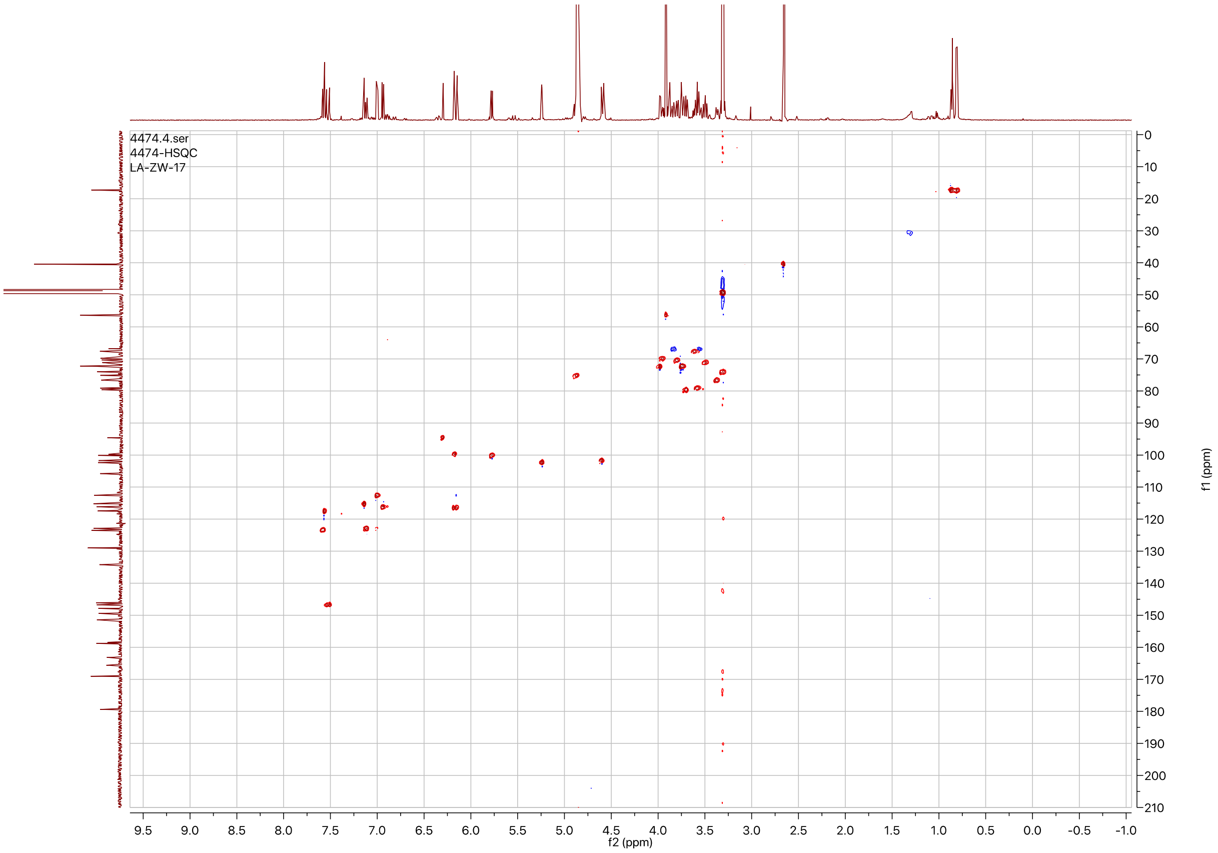

B

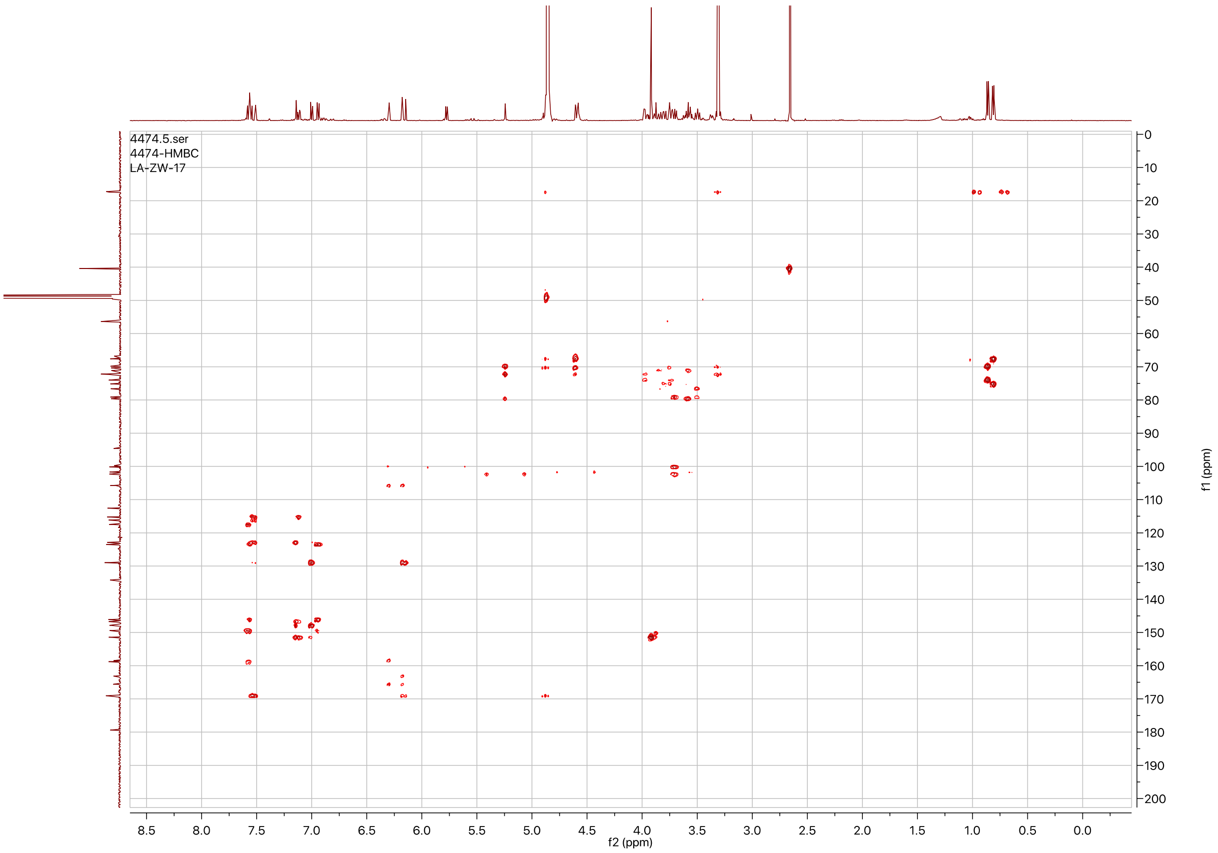

Figure S4. The HSQC (A) and HMBC (B) spectra of compound **8**.

References

1. Amer, M.; Abou-Shoer, M.; Abdel-Kader, M.; El-Shaibany, A.; Abdel-Salam, N., Alakloids and flavone acyl glycosides from *Acanthus arboreus*. *J Braz Chem Soc* **2004,** *15*, 262-266.
2. Itokawa, H.; Suto, K.; Takeya, K., Studies on a novel p-coumaroyl glucoside of apigenin and on other flavonoids isolated from patchouli (Labiateae). *Chem Pharmaceut Bull* **1981,** *29*, 254-256.
3. Ferreira, F. P.; de Oliveira, D. C. R., New constituents from Mikania laevigata Shultz Bip. ex Baker. *Tetrahedron Letters* **2010,** *51*, 6856-6859, 10.1016/j.tetlet.2010.10.102.
4. Budzianowski, J.; Skrzypczak, L., Phenylpropanoid esters from *Lamium album* flowers. *Phytochemistry* **1995,** 38, 997-1001, DOI: 10.1016/0031-9422(94)00727-B.
5. Zhang, H.; Rothwangl, K.; Mesecar, A. D.; Sabahi, A.; Rong, L.; Fong, H. H. S., Lamiridosins, hepatitis C virus entry inhibitors from *Lamium album*. *J Nat Prod* **2009,** *72*, 2158-2162, DOI: 10.1021/np900549e.
6. Kobayashi, S.; Mima, A.; Kihara, M.; Imakura, Y., Iridoid Glucosides from *Lamium amplexicaule*. *Chemical & Pharmaceutical Bulletin* **1986,** *34*, 876-880. <Go to ISI>://WOS:A1986A500600060
7. Calis, I.; Basaran, A. A.; Saracoglu, I.; Sticher, O.; Ruedi, P., Phlinosides A, B and C, three phenylpropanoid glycosides from *Phlomis linearis*. *Phytochemistry* **1990,** *29*, 1253-7, DOI: 10.1016/0031-9422(90)85436-J.
8. Yalçin, N. F.; Ersöz, T.; Bedir, E.; Şahpaz, S.; Bailleul, F.; Khan, I. A.; Dönmez, A. A.; Çalis, I., Phlinoside F, a new phenylethanoid glycoside from *Phlomis angustissima*. *Turk J Chem* **2005,** *29*, 417-423. www.researchgate.net/publication/297931384_Phlinoside_F_a_new_phenylethanoid_glycoside_from_Phlomis_angustissima (accessed on 16 September 2014)
9. Calis, I.; Basaran, A. A.; Saracoglu, I.; Sticher, O.; Ruedi, P., Phlinosides D and E, phenylpropanoid glycosides, and iridoids from *Phlomis linearis*. *Phytochemistry* **1991,** *30*, 3073-5, DOI: 10.1016/S0031-9422(00)98255-4.
